# Enhanced acetic acid stress tolerance and ethanol production in *Saccharomyces cerevisiae* by modulating expression of the de novo purine biosynthesis genes

**DOI:** 10.1186/s13068-019-1456-1

**Published:** 2019-05-10

**Authors:** Ming-Ming Zhang, Liang Xiong, Ya-Jie Tang, Muhammad Aamer Mehmood, Zongbao Kent Zhao, Feng-Wu Bai, Xin-Qing Zhao

**Affiliations:** 1 State Key Laboratory of Microbial Metabolism, Joint International Research Laboratory of Metabolic & Developmental Sciences, School of Life Sciences and Biotechnology, Shanghai Jiao Tong University, Shanghai, 200240 China; 2 Key Laboratory of Fermentation Engineering (Ministry of Education), Hubei Provincial Cooperative Innovation Center of Industrial Fermentation, Hubei Key Laboratory of Industrial Microbiology, Hubei University of Technology, Wuhan, 430068 China; 3 School of Life Science and Biotechnology, Dalian University of Technology, Dalian, 116024 China; 4 Department of Biotechnology, Dalian Institute of Chemical Physics, Chinese Academy of Sciences, Dalian, 116023 China; 5 State Key Laboratory of Microbial Technology, Shandong University, Qingdao, 266237 China; 6 Department of Bioinformatics & Biotechnology, Government College University Faisalabad, Faisalabad, 38000 Pakistan

**Keywords:** *Saccharomyces cerevisiae*, Yeast stress tolerance, de novo purine biosynthesis, *ADE17*, Global amino acid profiles

## Abstract

**Background:**

Yeast strains that are tolerant to multiple environmental stresses are highly desired for various industrial applications. Despite great efforts in identifying key genes involved in stress tolerance of budding yeast *Saccharomyces cerevisiae*, the effects of de novo purine biosynthesis genes on yeast stress tolerance are still not well explored. Our previous studies showed that zinc sulfate addition improved yeast acetic acid tolerance, and key genes involved in yeast stress tolerance were further investigated in this study.

**Results:**

Three genes involved in de novo purine biosynthesis, namely, *ADE1*, *ADE13*, and *ADE17*, showed significantly increased transcription levels by zinc sulfate supplementation under acetic acid stress, and overexpression of these genes in *S. cerevisiae* BY4741 enhanced cell growth under various stress conditions. Meanwhile, ethanol productivity was also improved by overexpression of the three *ADE* genes under stress conditions, among which the highest improvement attained 158.39% by *ADE17* overexpression in the presence of inhibitor mixtures derived from lignocellulosic biomass. Elevated levels of adenine-nucleotide pool “AXP” ([ATP] + [ADP] + [AMP]) and ATP content were observed by overexpression of *ADE17*, both under control condition and under acetic acid stress, and is consistent with the better growth of the recombinant yeast strain. The global intracellular amino acid profiles were also changed by overexpression of the *ADE* genes. Among the changed amino acids, significant increase of the stress protectant γ-aminobutyric acid (GABA) was revealed by overexpression of the *ADE* genes under acetic acid stress, suggesting that overexpression of the *ADE* genes exerts control on both purine biosynthesis and amino acid biosynthesis to protect yeast cells against the stress.

**Conclusion:**

We proved that the de novo purine biosynthesis genes are useful targets for metabolic engineering of yeast stress tolerance. The engineered strains developed in this study with improved tolerance against multiple inhibitors can be employed for efficient lignocellulosic biorefinery to produce biofuels and biochemicals.

**Electronic supplementary material:**

The online version of this article (10.1186/s13068-019-1456-1) contains supplementary material, which is available to authorized users.

## Background

The budding yeast *Saccharomyces cerevisiae* is widely used as a cell factory for production of biofuels and biochemicals. Yeast cells are subjected to various adverse conditions during industrial applications, and improving tolerance of the yeast cells to multiple environmental stresses benefits efficient bioproduction [1]. Therefore, studies on the underlying mechanisms of yeast stress tolerance and strategies to develop robust strains that are tolerant to various stresses have received continuous attention [2–7]. Lignocellulosic biomass, such as agricultural and forest residues, is abundant in nature, and is widely studied as promising renewable feedstocks to produce biofuels and biochemicals [2, 3]. However, various inhibitors, including acetic acid, furfural, formic acid, and 5-hydroxymethyl-2-furfural (5-HMF), may be released during the decomposition process of lignocellulosic feedstocks to obtain fermentable sugars, and the bioconversion efficiency of yeast strains can be severely compromised [8]. Therefore, development of robust yeast strains that are tolerant to various stress conditions is highly desired for lignocellulosic biorefinery.

Among the lignocellulosic hydrolysate-derived inhibitors, acetic acid is a major inhibitor and is commonly present in various hydrolysates [8]. Acetic acid at toxic level inhibits yeast cell growth by impeding the metabolic functions through intracellular acidification [9]. Moreover, repression of nutrient and energy utilization under acetic acid stress also leads to growth inhibition [10]. High concentration of acetic acid also causes the accumulation of reactive oxygen species (ROS) [11, 12], thereby leads to oxidative damage. Great efforts have been made to improve yeast acetic acid tolerance by evolutionary engineering [13] or metabolic engineering [14–17], and studies on the underlying mechanisms of acetic acid toxicity not only provide insights in yeast stress response, but also benefit strain development by identification of novel candidate genes for metabolic engineering of yeast stress tolerance [7, 10, 14, 17–20].

Zinc ion is an essential nutrient and acts as structural and catalytic co-factor for many important proteins [21, 22]. The intracellular zinc homeostasis is important for normal function of cells, which is mainly regulated by a metalloregulatory protein Zap1p [23]. Studies in our group showed that zinc status plays important roles in yeast stress tolerance. For example, zinc sulfate addition increased cell viability and ethanol production during high gravity ethanol fermentation [24]. Improved growth and ethanol fermentation performance under acetic acid stress by zinc supplementation was also observed [12, 25]. In our previous studies, changes in alanine metabolism and transcription levels of membrane transporters were revealed by zinc supplementation in the presence of acetic acid stress, and deletion of the zinc-responsive transporter *ADY2* enhanced ethanol production [12, 17]. It is of great interest to explore more critical molecular targets by studying the underlying mechanisms by which zinc sulfate improved yeast stress tolerance.

Purine metabolism is important for cellular function of *S. cerevisiae* as well as in virtually every living organisms. The ADEnine requiring (*ADE*) genes, including *ADE1*, *ADE13,* and *ADE17*, among others, participate in the de novo purine biosynthesis pathway yielding inosine monophosphate (IMP) and adenosine 5′-monophosphate (AMP) [26], and IMP is the first purine nucleotide in the pathway which can be converted to AMP and guanosine 5′-monophosphate (GMP). Purine nucleotides are the major carries of cellular energy, and are also involved in various enzymatic reactions for normal cellular functions [27]. Among the *ADE* genes, *ADE17* encodes the enzyme which contains both 5-aminoimidazole-4-carboxamide ribonucleotide (AICAR) transformylase and IMP cyclohydrolase activities. Significant activation of *ADE17* transcription in response to short-time acetic acid shock treatment was reported [28]. Meanwhile, transcription of *ADE17* was elevated in response to spermidine addition, and overexpression of *ADE17* enhanced tolerance to furfural and 5-HMF in *S. cerevisiae* D452-2 [4]. However, the important roles of other de novo purine metabolism genes in prolonged stressful conditions, as well as the underlying mechanisms are still not clear.

In the present study, we report positive roles of three *ADE* genes that are responsible for de novo purine metabolism in the zinc-mediated improved stress tolerance in *S. cerevisiae*. The engineered *S. cerevisiae* strains exhibited enhanced tolerance to various inhibitory conditions. Furthermore, analysis of the engineered yeast strains revealed variations in cellular-nucleotide pool and key amino acids. Our results emphasized the importance of genes involved in de novo purine metabolism in metabolic engineering of yeast stress tolerance, and provided basis for developing efficient strains for efficient biorefinery of lignocellulosic biomass.

## Results

### Transcriptome analyses revealed remodeling of purine metabolism upon zinc sulfate supplementation in the presence of acetic acid

**Fig. 1:**
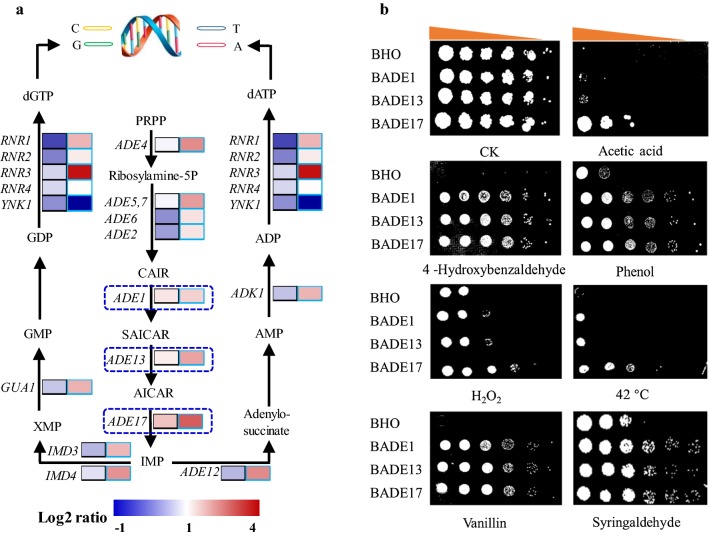
Differential transcription of genes involved in purine metabolism under acetic acid stress by addition of zinc sulfate. Comparative transcriptomic analyses (**a**) were performed with yeast cells harvested at log phase (left column) as well as the same timepoints (right column). Comparison of stress tolerance of the engineered strains BADE1, BADE13 and BADE17 with BHO under various conditions (**b**)

In our previous studies, global gene transcription of the flocculating yeast SPSC01 under acetic acid stress with and without zinc sulfate supplementation was compared when cells were collected at the same timepoint (72 h after inoculation) [17]; however, the growth states of the strains were different at the same timepoint. For the zinc-addition culture, yeast cells attained exponential phase at 72 h; however, it took the control culture 90 h to reach the exponential phase. Hence, we re-analyzed the RNA-seq data by comparing global transcription at the exponential phase in the current work. The results showed that among all the differentially transcribed genes, genes involved in nucleotide metabolism, mainly purine biosynthesis, were enriched (Fig. 1a). Several *ADE* genes, especially *ADE1*, *ADE13*, and *ADE17* that participate in de novo purine synthesis, showed significant changes with zinc sulfate addition. Especially, transcription activation of *ADE17* by zinc supplementation was observed when compared both at log phase and at the same fermentation time (Fig. 1a). In addition, enhanced transcription of *ADE17* in different yeast strains (BY4741, SPSC01, and Ethanol Red) when grown in the presence of acetic acid was also revealed by real-time quantitative PCR (RT-qPCR) analysis (Additional file 1: Fig. S1). To further investigate the roles of these genes in yeast stress tolerance, the three *ADE* genes were overexpressed in *S. cerevisiae* BY4741, and the recombinant strains were named BADE1, BADE13, and BADE17, respectively. When cell growth of the yeast strains was evaluated, it was revealed that the recombinant strains were more resistant to various inhibitors present in cellulosic hydrolysate, including acetic acid, hydroxybenzaldehyde, phenol, vanillin, and syringaldehyde (Fig. 1b). Whereas under non-stressed growth conditions, all the engineered strains showed almost identical growth when compared with the control strain BHO through the spot assay (Fig. 1b). On the other hand, only overexpression of *ADE17*, but not *ADE1* and *ADE13*, resulted in improved growth under osmotic stress with sodium chloride. Robust growth of the recombinant strains was also observed in the presence of propionic acid, and BADE17 showed the best growth under various stress conditions comparing with that of BADE1 and BADE13 (Fig. 1b; Additional file 1: Fig. S2), suggesting that *ADE17* plays the most critical roles in stress tolerance among the three *ADE* genes. We proved here that overexpression of the *ADE* genes endowed yeast cells with enhanced growth performance under prolonged treatment of multiple stresses. The binding sites of several stress responsive transcription factors were found in the promoter regions of the *ADE* genes (Additional file 1: Fig. S3), suggesting that these *ADE* genes are stress responsive genes, and are potentially subjected to regulation by different regulators.

### Effects of overexpressing *ADE* genes on fermentation performance of *S. cerevisiae* under stresses conditions

**Fig. 2:**
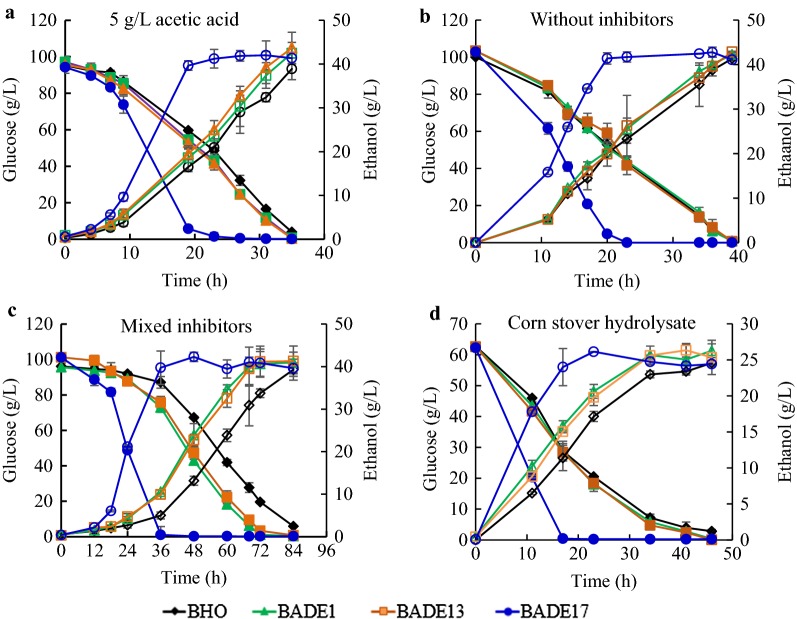
Impact of the *ADE* genes overexpression on ethanol fermentation. Ethanol fermentation was performed in bioreactor with 5 g/L acetic acid (**a**) or mixed inhibitors (**c**) supplemented, and pH was controlled at 4.5, and in flask without external inhibitors addition (**b**) and with corn stover hydrolysate (**d**). Results are the average of three independent experiments

Time courses of ethanol fermentation by the recombinant strains are illustrated in Fig. 2. The maximum biomass of the engineered strains BADE1, BADE13, and BADE17 increased 9.34%, 4.76%, and 38.80%, respectively, in contrast to the control strain BHO, under acetic acid stress (in the bioreactor, pH 4.5) (Additional file 1: Fig. S4A). Strain BADE17 utilized almost all of the glucose within 27 h, and strains BADE1 and BADE13 required 35 h to consume the same amount of the glucose. However, the control strain BHO could not consume the provided glucose even in 35 h, leaving 3.95 g/L glucose in the medium. Meanwhile, the ethanol productivity of strains with *ADE1*, *ADE13,* and *ADE17* overexpression was 1.21, 1.20, and 1.55 g/L/h, respectively, while for the control strain, it was only 1.11 g/L/h (Fig. 2a). Improved fermentation performance was also observed when fermentation was performed under 3.6 g/L (in flask, initial pH 3.7) acetic acid condition (Additional file 1: Fig. S4B). In addition, different from BADE1 and BADE13, strain BADE17 also exhibited improved fermentation performance and better growth than the control strain BHO under the condition without any stress (Fig. 2b; Additional file 1: Fig. S4C).

Ethanol fermentation performance was further evaluated under simulated hydrolysate and authentic corn stover hydrolysate. When tested using the simulated lignocellulosic hydrolysate, the fermentation time of the engineered strains BADE1, BADE13, and BAD17 was 72, 84, and 36 h, respectively (Fig. 2c), whereas there was still 5.85 g/L residual glucose for the control strain BHO even after 84 h fermentation. Meanwhile, higher biomass and ethanol productivity were also achieved by the *ADE* genes overexpression strains (Additional file 1: Fig. S4D). In addition, rapid decrease of furfural and 5-HMF concentrations was observed by overexpression of *ADE17* in the simulated hydrolysate (Additional file 1: Fig. S5), which may contribute to the highly efficient fermentation performance. Furthermore, clear improvement of ethanol production by *ADE17* overexpression was observed from the corn stover hydrolysate (Fig. 2d). No significant change of fermentation performance of BADE1, BADE13 was observed from the corn stover hydrolysate, possibly due to lower concentrations of inhibitors and glucose. The results of ethanol fermentation in bioreactor by the engineered strains are summarized in Table 1. These results suggested that overexpression of the *ADE* genes protected yeast strains against various stress conditions, and *ADE17* has the most significant effects to promote growth and fermentation under multi-stress conditions.

**Table 1:** Ethanol production performance of the recombinant yeast strains under various conditions

Parameter	5 g/L acetic acid				Mixed inhibitors			
	BHO	BADE1	BADE13	BADE17	BHO	BADE1	BADE13	BADE17
*t* (h)	35	35	35	27	84	72	84	36
*S*_R_ (g/L)	3.95	0.73	0.42	0.53	5.85	0.92	0.80	0.84
*E*_p_ (g/L)	38.90	42.50	42.10	41.86	37.69	40.63	41.28	41.74
*q* (g/L/h)	1.111	1.214	1.203	1.550	0.449	0.564	0.491	1.159
*Y*_E*/*S_ (g/g)	0.422	0.432	0.436	0.440	0.398	0.407	0.408	0.412

*t*: fermentation time; *S*_R_: residual glucose; *E*_P_: ethanol produced; *q*: ethanol productivity; *Y*_E*/*S_: ethanol yield, g(ethanol)/g(glucose/sugars)

### Influence of *ADE* genes on intracellular ROS accumulation and transcription of the key genes involved in stress tolerance

**Fig. 3:**
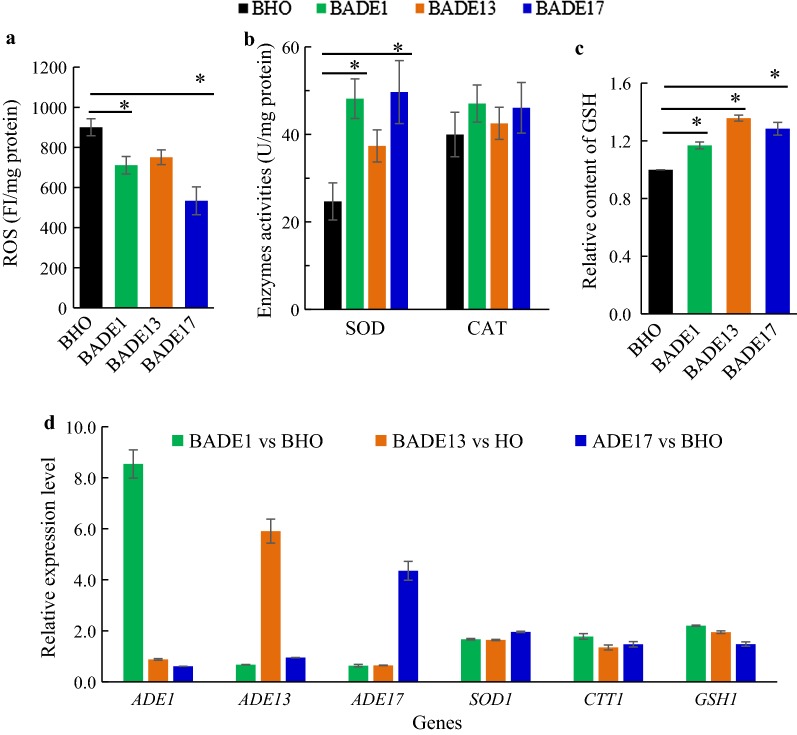
Influence of the *ADE* genes overexpression on ROS response and expression of key genes under acetic acid condition. ROS accumulation (**a**), activities of antioxidant enzymes (**b**), GSH content (**c**), and transcription levels of stress tolerance-related genes (**d**) were detected in strains BADE1, BADE13, BADE17, and BHO. Gene *ACT1* expression was used as a reference in the RT-qPCR analysis. Results are the average of three independent experiments. *p value < 0.05 and **p value < 0.01 in significance analysis using *t* test

The molecular basis underlying improved stress tolerance by overexpression of the *ADE* genes was further investigated. ROS are generated during normal metabolic processes or in response to some external environmental stress, especially under aerobic fermentation, which might damage cellular components [29]. The intracellular ROS accumulation was decreased by 21.04%, 16.61%, and 40.74% by *ADE1*, *ADE13,* and *ADE17* overexpression under acetic acid stress, respectively (Fig. 3a). Activities of the antioxidant enzymes SOD and CAT were further analyzed under acetic acid stress. The activities of SOD for the recombinant strains BADE1, BADE13, and BADE17 were significantly higher than that of the control strain, which were 95.05%, 51.28%, and 101.13%, respectively (Fig. 3b). However, no significant increase in CAT activity was detected in all the recombinant strains. Besides, a significant increase of glutathione (GSH) content was detected in all the engineered strains compared to that of the control strain (Fig. 3c), which contributes to ROS detoxification.

RT-qPCR analyses were further performed. As shown in Fig. 3d, the transcription levels of the *ADE* genes obviously were up-regulated in the corresponding engineered strains under acetic acid stress. However, there was no interactive impact of the *ADE* genes with each other. The key genes involved in oxidative stress tolerance, namely, *SOD1*, *CTT1,* and *GSH1*, showed elevated transcription levels in the engineered strains comparing with that of the control strain, suggesting that up-regulation of the *ADE* genes affected key genes in the endogenous antioxidant system.

### Influence of *ADE* genes overexpression on the intracellular adenine-nucleotide pool of the engineered yeast strains

**Fig. 4:**
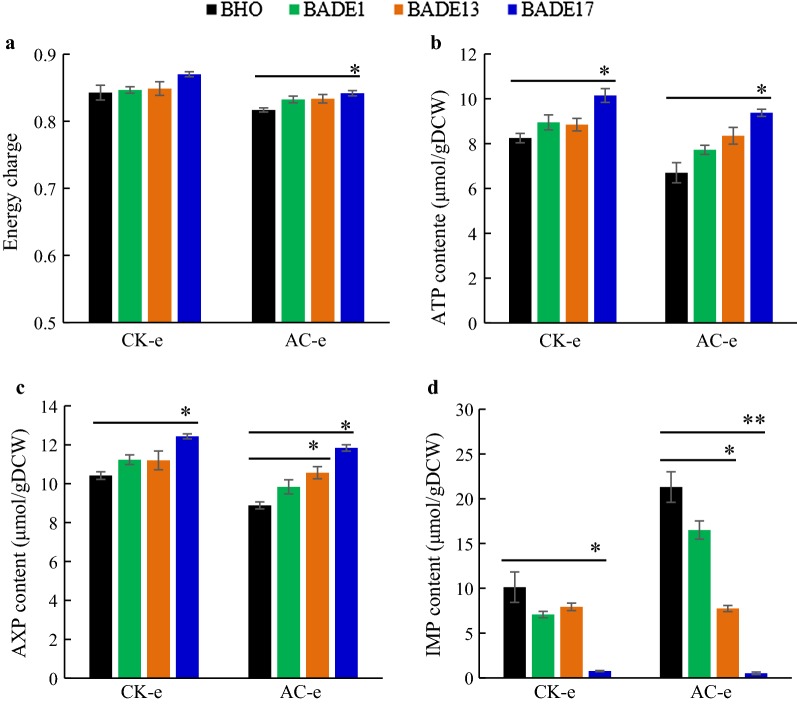
Effect of the *ADE* genes overexpression on intracellular adenine-nucleotide pool. Samples of the recombinant strains and control strain BHO were harvested at exponential phase for determination of energy charge (**a**), ATP concentration (**b**), total AXP content (**c**), and IMP concentration (**d**), respectively. CK represents under control condition, AC represents the acetic acid stress, and e represents samples were harvested at the exponential phase. Each value represented the mean value of three independent replicates. *p value < 0.05 and **p value < 0.01 in significance analysis using *t* test

One toxic effect of weak acid stress is decrease of energy charge [30] and intracellular ATP level [3, 31]. Therefore, it is of interest whether overexpression of the *ADE* genes affects ATP level and energy charge. As shown in Fig. 4, under non-stressed conditions, there was no obvious difference of energy charge among the control strain BHO and the recombinant strains overexpressing the *ADE* genes at the exponential phase (Fig. 4a). However, under acetic acid stress, increased energy charge and improved intracellular ATP content were observed by overexpression of *ADE17* when compared to that of the control strain BHO (Fig. 4a, b). In addition, increased ATP content was also observed by overexpression of *ADE17* at the stationary growth phase (Additional file 1: Fig. S6B).

Overexpression of *ADE1, ADE13,* and *ADE17* genes also increased the adenine-nucleotide pool “AXP” ([ATP] + [ADP] + [AMP] = [AXP]) [32], which were 10.76%, 18.91%, and 33.29% higher, respectively, than that of the control strain at the exponential phase under acetic acid stress (Fig. 4c). Meanwhile, increased AXP pool was also observed at the stationary phase in all the recombinant strains (Additional file 1: Fig. S6C). In addition, significantly decreased IMP content, which is the precursor of adenine nucleotide, was observed by *ADE17* overexpression (Fig. 4d and Additional file 1: S6D). Increased AXP pool and decreased IMP content were also apparent by overexpression of *ADE13* under acetic acid stress, but no significant change in intracellular AXP pool and energy charge was observed by *ADE1* overexpression.

### Influence of *ADE* genes overexpression on intracellular amino acid profile

**Fig. 5:**
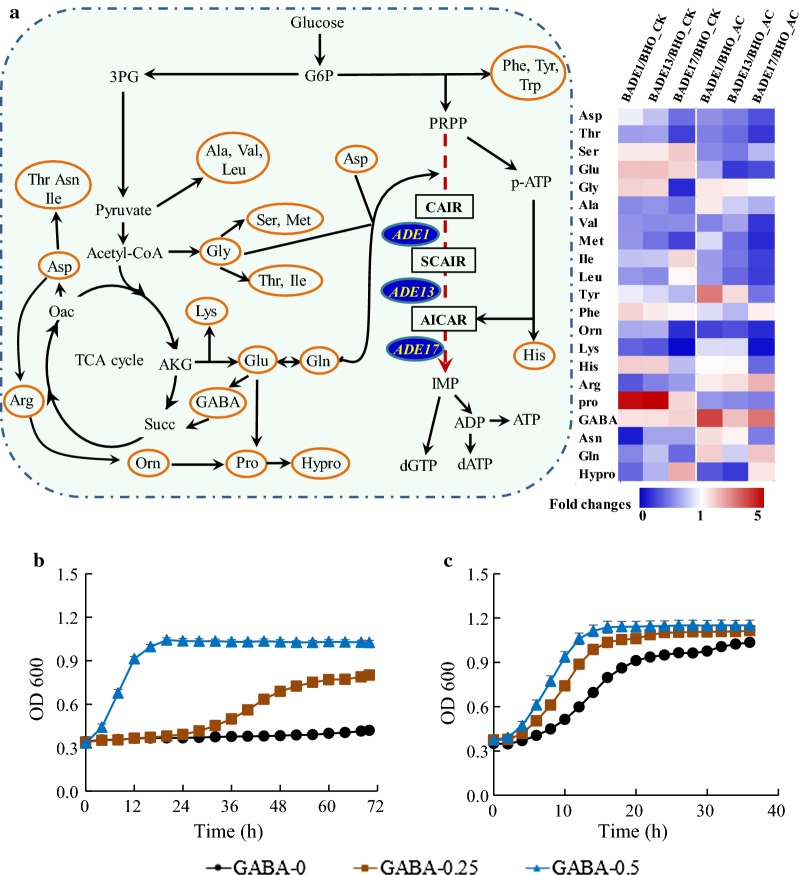
Effect of the *ADE* genes overexpression on the relative abundance of intracellular amino acid. The relative abundance was calculated by normalization of the concentration of the control strain BHO under control (CK) or acetic acid (AC) conditions (**a**). Yeast cells were cultured in the YPD medium with GABA (0, 0.25 and 0.5 g/L) addition in the presence of 5.0 (**b**) or 3.6 (**c**) g/L acetic acid. Results are the average of two runs

Purine metabolism is closely related to metabolism of specific amino acids through the co-precursor 5-phosphoribosyl diphosphate (PRPP), co-metabolic intermediate AICAR and the common transcription factors Bas1p/Bas2p [33, 34]. Hence, the global amino acid profiles in the recombinant strains were examined. Under non-stressed conditions, relative levels of amino acids Glu and GABA were enhanced in the recombinant strains overexpressing the three *ADE* genes when compared with BHO. In contrast, the contents of Thr, Ala, Val, Met, Orn, Lys, Arg, Gln, and Asn were decreased in all the three recombinant strains (Fig. 5a). The other amino acids showed different variable trends in the engineered strains. These results indicated that genetic manipulation of purine metabolism modified amino acid metabolism. When compared the effect of the different *ADE* genes, overexpression of *ADE17* showed different amino acid concentration profiles from that of *ADE1* and *ADE13*, and similar profiles of endogenous amino acid were observed when *ADE1* and *ADE13* were overexpressed. Interestingly, different amino acid profiles were observed when yeast cells were investigated under acetic acid stress. The levels of amino acids of Arg and GABA remarkably increased in all the engineered strains under acetic acid stress, whereas decreased levels of Asp, Ser, Glu, Ile, Leu, Orn, and Pro were apparent. These results indicated that the amino acids were reprogrammed by overexpression of the *ADE* genes when yeast cells are subjected to acetic acid stress. Meanwhile, there was a variable trend of change in amino acids contents in the engineered strains under acetic acid stress, indicating that the change of amino acid was affected by both the manipulated genes and the environmental conditions.

Among the amino acids that we detected in this study, intracellular GABA content always showed a significant increase in the recombinant strains, especially under acetic acid stress. We proposed that the increase in the intracellular GABA content by modulating purine metabolism conferred protection against the acetic acid toxicity. Hence, growth performance analysis was carried out to evaluate the effects of amino acids addition on acetic acid tolerance of yeast cells. As shown in Fig. 5b, the remarkably beneficial effect of GABA on acetic acid tolerance was observed. The lag phase was shortened to 24 h with 0.25 g/L GABA addition under 5 g/L acetic acid stress when compared with that of the control group without addition. Furthermore, almost no obvious lag phase was observed when the concentration of added GABA raised to 0.5 g/L. During the culture, the maximum OD_600_ were 0.42, 0.80, and 1.03, respectively, when 0, 0.25, and 0.5 g/L GABA were supplied. There were close interactions between the metabolism of GABA, Glu, and Gln. Hence, we assumed that the Glu and Gln addition might improve intracellular GABA content, leading to improved tolerance to acetic acid. Indeed, addition of Glu (1.5 g/L) and the Gln (0.5 and 1.5 g/L) notably improved growth performance in the presence of 5 g/L acetic acid (Additional file 1: Fig. S7A, B). In addition, the beneficial effects of GABA, Glu and Gln addition on cells grown in presence of 3.6 g/L acetic acid were also observed (Fig. 5c; Additional file 1: Fig. S7C, D).

Intracellular zinc content of *S. cerevisiae* BY4741 was detected under both acetic acid stress and stress-free conditions, and it was found that under acetic acid stress, the intracellular zinc content was 49% lower than that in the control condition, suggesting a zinc deficiency by acetic acid stress. The zinc-responsive regulator Zap1p binds zinc-responsive element (ZRE) in the promoter region of its target genes, and *ADE17* harbors a ZRE in its promoter region [23]. To check whether the function of *ADE17* is directly related to zinc response, we performed mutation of the ZRE, and compared acetic acid tolerance of the mutant with that of the parent strain. The results showed that the mutation led to decreased acetic acid tolerance (Additional file 1: Fig. S8), suggesting a direct relation of zinc with the function of *ADE17* in maintaining yeast cell growth under acetic acid stress.

## Discussion

**Fig. 6:**
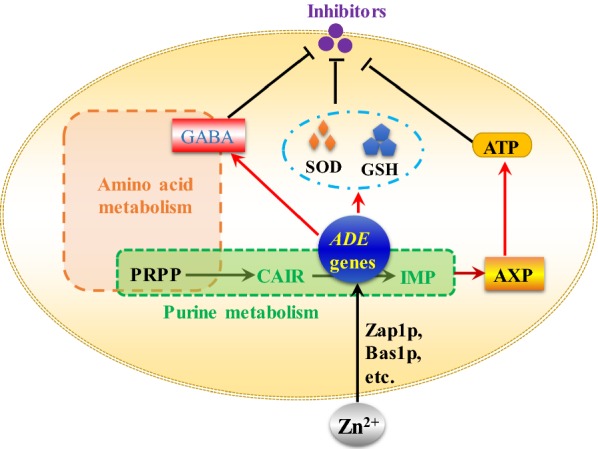
Hypothesis on the mechanisms of enhanced tolerance to multiple inhibitors by overexpression of the *ADE* genes. Zinc ion addition upregulates transcription level of the *ADE* genes under acetic acid stress condition through transcription factors, including Zap1p, Bas1p, and others. Elevated transcription of the *ADE* genes results in enhanced adenine-nucleotide pool, higher antioxidant capacity, and improved GABA content, which contribute to enhanced tolerance to inhibitors

Zinc ion is an important nutrient for yeast cells, and the zinc status is especially important under stress conditions [12, 24, reviewed in 22]. Therefore, studies on the mechanisms underlying improved stress tolerance by manipulating zinc status are of significance for efficient bioproduction. Different from the previous report, where changes in *ADE* gene transcription were revealed by high zinc levels [35], our studies here showed that suitable zinc concentration affected *ADE* gene transcription under acetic acid stress. The change of intracellular zinc concentration by acetic acid stress suggests that zinc status plays important role in acetic acid stress tolerance. Furthermore, we found that mutation of the ZRE in the promoter region of *ADE17* led to decreased growth ability under acetic acid stress. We speculated that response to intracellular zinc ion concentration by *ADE17* is important for regulation of yeast stress tolerance, and during this process, Zap1p is one of the critical transcription regulator. Meanwhile, other transcription factors affected by zinc addition may also participated in global gene transcription changes under acetic acid stress, and accompanied with the modulation of intracellular zinc state; transcription of the *ADE* genes involved in de novo purine biosynthesis was affected. Subsequently, we proved that three *ADE* genes, especially *ADE17*, which are involved in de novo purine biosynthesis, are important for yeast stress tolerance. The mechanisms of the improved stress tolerance involve antioxidant system, and changes in nucleotide pool and amino acid contents. The proposed mechanisms of improved stress tolerance by *ADE* genes overexpression are presented in Fig. 6. Our results emphasized that the importance of genes involved in de novo purine metabolism in metabolic engineering of yeast stress tolerance, and it will be interesting to examine the effects of co-expression of the three *ADE* genes on yeast stress tolerance.

In response to oxidative stress in yeast cells that is induced by acetic acid, enzymatic system (antioxidant enzymes), and non-enzymatic defense system (GSH and other small molecules) are employed [36]. Decreased ROS level was observed in the engineered strains, which was consistent with the increased SOD activities. However, no obvious difference of CAT enzyme among the engineered strains compared with that of the control, indicating that these genes endowed oxidation tolerance mainly through activation of SOD, which is consistent with the up-regulation of *SOD1*. Meanwhile, an obvious increase of intracellular GSH, as well as elevated transcription of *GSH1*, was observed in the three engineered strains, which contributed to protecting yeast cells against oxidative stress [37]. Higher GSH content was found also in the more tolerant yeast cells with zinc addition than that without zinc addition [12]. Taken together, detoxification of intracellular ROS by the elevated expression of the *ADE* genes contributes to improved stress tolerance.

We also revealed changes in ATP and nucleotide pool by overexpression of the *ADE* genes. To maintain the homeostasis of intracellular environment under acetic acid stress, H^+^-ATPase (proton-translocating ATPase), responsible for protons expulsion, and ABC (ATP-binding cassette) transporter, responsible for acetate expulsion, were activated in an ATP-dependent manner [30, 38]. However, these processes would lead to depletion of intracellular ATP, which was indicated by the fact that a 40% reduction of intracellular ATP content was detected in the presence of 220 mM acetic acid [30]. Hence, we concluded that the increase of intracellular ATP content resulting from overexpression of *ADE17* exerted a positive impact on yeast stress tolerance. The nucleotide AXP concentration may be dropped in response to changing growth conditions due to the insufficient supply of phosphoric acid groups [32]. The engineered strain BADE17 showed higher content of AXP pool and significantly decreased IMP content, and we assumed that overexpression of *ADE17* led to return of the intracellular adenine-nucleotide pool and relief of energy stress. Further studies are worthwhile to investigate the underlying mechanisms.

Amino acid metabolism is of great importance for yeast metabolism and yeast stress tolerance [39, 40]. Here, we identified that zinc sulfate in the medium affected genes involved in purine metabolism, especially *ADE17*, and overexpression of three *ADE* genes leads to variable changes in global amino acid metabolism. Although it is known that some amino acids, including Gln, Glu, and His, are closely related to purine metabolism [41], so far no study was performed on changes of purine metabolism by key purine metabolism genes together with global amino acid metabolism. We found that yeast intracellular amino acid concentrations profiles were changed by acetic acid stress, and overall *ADE17* overexpression leads to more severe decreased amino acid biosynthesis than that of *ADE1* and *ADE13*. In a recent report, response of yeast amino acid metabolome to systematic gene deletion was reported [42], which revealed that a major part of coding genes are involved in gene–metabolism interactions. Our current study is the first to present the global amino acid profiles by overexpression of the *ADE* genes. In addition, we revealed changes of the global amino acid profiles under low-dose long-term acetic acid stress, which was different from that in the previous study under acetic acid shock stress [43]. In the previous study, dramatic depletion of all the detected amino acids was observed by acetic acid treatment, indicating that acetic acid stress led to amino acid starvation [43]. Our results added further evidences that overexpression of the *ADE* genes regulate amino acid metabolism differently under acetic acid stress when compared with that without stress.

GABA shunt pathway plays an important role in succinate metabolism, which is involved in the formation of succinate [44]. Increased intracellular succinate content was observed in the engineered strains (Additional file 1: Fig. S9), which is consistent with the active GABA metabolism. Moreover, GABA exerts important roles in the control of cytosolic pH, restricting ROS production and adaptation to heat stress [44]. It was reported that yeast strains with higher GABA content exhibited increased tolerance to FAP (furfural, acetic acid, and phenol) and combined lignocellulosic-derived inhibitors [45, 46]. Meanwhile, deletion of *UGA1* that is involved in GABA metabolism reduced growth ability of yeast in the presence of FAP [40]. However, the effect of GABA on long-time moderate acetic acid stress tolerance in *S. cerevisiae* has not been reported. In this study, we demonstrated that GABA supplementation exerted a positive effect on acetic acid tolerance. These results suggest that improved acetic acid tolerance may be achieved by manipulating biosynthesis of key amino acids including GABA biosynthesis.

## Conclusions

In this study, enhanced growth ability and ethanol fermentation performance under stress conditions was achieved by overexpression of *ADE1*, *ADE13* and *ADE17*. Overexpression of *ADE17* showed the most significant effect among the three *ADE* genes. Elevation of the adenine-nucleotide pool, decreased ROS accumulation, and enhanced GABA content were observed by overexpression of *ADE17*, which may contribute to the improved growth and ethanol fermentation performance. The results in this study reveal that de novo purine metabolism is a useful target for metabolic engineering of yeast to develop robust yeast factories.

## Materials and methods

### Strains and culture media

*Escherichia coli* DH5α was used as a host strain for gene cloning and genetic manipulation. *S. cerevisiae* SPSC01 and *S. cerevisiae* BY4741 (BY for short in the following text) were used as host strains, and all strains employed in this study are listed in Additional file 1: Table S1.

*Escherichia coli* DH5α was cultivated in Luria–Bertani (LB) medium and yeast strains were cultivated in YPD medium as described elsewhere [15]. For ethanol fermentation, the fermentation medium (100 g/L glucose, 4 g/L yeast extract, and 3 g/L peptone) were used, and 5 g/L acetic acid was added to evaluate the effect of acetic acid stress. The simulated hydrolysate was prepared with the same composition of the fermentation medium, and inhibitor mixture (6.5 g/L acetic acid, 0.8 g/L furfural, 0.5 g/L formic acid, and 0.6 g/L 5-HMF) was added. The corn stover hydrolysate (containing 62.55 g/L glucose, 23.31 g/L xylose, 3.74 g/L acetic acid, 0.33 g/L furfural, 0.22 g/L 5-HMF, and 1.02 g/L formic acid) was also used, and 4 g/L yeast extract and 3 g/L peptone were supplemented in the hydrolysate for ethanol fermentation.

### RT-qPCR analysis

Total RNA was extracted from yeast cells and was reversely transcribed into cDNA using a PrimeScript RT Reagent Kit (TaKaRa, Dalian, China), and was used for RT-qPCR analysis. The mRNA transcript levels were normalized using *ACT1* as a reference. Relative expression levels were determined by the 2^−ΔΔ^Ct method [47]. All RT-qPCR primers used in this study are listed in Additional file 1: Table S2.

### RNA-sequencing data analysis

RNA-seq data from the previous study [17] were re-analyzed using the data at the exponential phase (72 h for the zinc sulfate addition sample and 90 h for the control sample). Differentially expressed genes were selected using two criteria: *P *< 0.05 in *t* test; and |log2 (Fold change)| > 1. Enrichment of functional categories among differentially expressed genes was examined using the MIPS Function Catalog (http://mips.gsf.de). Specific gene functions were analyzed based on the information from the *Saccharomyces* Genome Database (SGD) (http://www.yeastgenome.org), and biological pathways were analyzed based on KEGG database (http://www.kegg.jp).

### Construction of the recombinant yeast strains

The laboratorial BY strain was engineered to overexpress *ADE1, ADE13,* and *ADE17* genes. The gene *ADE1* was amplified (ADE1F/R) by Polymerase Chain Reaction (PCR) using the genomic DNA of *S. cerevisiae* S288c as a template. The amplified sequences were inserted into an integration plasmid pHO to obtain expression plasmid, pHO-ADE1. Yeast transformation and selection of the transformants were performed using the methods described previously [15] and the engineered strains were named BADE1, BADE13 and BADE17, respectively. The empty plasmid pHO was transformed into BY strain to obtain the control strain BHO.

Mutation of the ZRE in the promoter region of *ADE17* in BY strain was introduced using the CRISPR/Cas9 system as described previously [48, 49], resulting in the engineered strain ADE17_mZRE. pRS42H_gRNA_ADE17zre containing the guide RNA (gRNA) targeting to the promoter region of *ADE17* was constructed with the modified pRS42H_gRNA_scaffold plasmid and the chemically synthesized 24-nt primer pair (gRNA_ADE17-F/R), and the annealed primers were ligated into the plasmid within the two anti-parallel *Bsa*I restriction sites. The donor DNA (donor_ADE17zre) was synthesized as a gene block, with the original ZRE (5′-A**CC**TTTAGTGT-3′) in *ADE17* promoter region replaced by a non-consensus sequence (5′-A**AA**TTTAGTGT-3′). Briefly, plasmid Cas9-NAT (purchased from Addgene, catalog #64329) carrying the Cas9 cassette was firstly transformed into strain BY to construct BYCas9. Then, the plasmid pRS42H_gRNA_ADE17zre together with donor_ADE17zre was co-transformed into BYCas9 to construct ADE17_mZRE. Transformants were selected on YPD medium containing 100 μg/mL nourseothricin and 200 μg/mL hygromycin B. The correct mutants were confirmed by PCR with the primer pair ADE17-C-F/R and subsequent sequencing. All primers used for strains and plasmids construction in this study are listed in Additional file 1: Table S3.

### Ethanol fermentation and metabolites analyses

Ethanol fermentation was performed in a bioreactor (KF-2.5L, KoBio Tech, South Korea) as described previously [15]. Growth media was supplemented with zinc sulfate at the concentration of 0.03 g/L when required. Yeast growth was monitored by measuring optical density (OD) with a spectrophotometer at λ_600nm_. Glucose, ethanol, succinic acid, acetic acid, glycerol, formic acid, furfural, and 5-HMF concentrations in the fermentation broth were analyzed simultaneously by HPLC system with RI- and UV detector (Waters e2695, Waters, MA, USA). The column that used for components separating was the same with described previously [15]. The mobile phase was 4 mM H_2_SO_4_ at a flow rate of 0.6 mL/min, and the detection temperature is 50 °C for RI-detector and 65 °C for the column. Samples were analyzed in triplicate, and the mean values were calculated.

### Stress tolerance, chemical analysis, and enzyme activity assays

Spot assay was performed as described in our previous study [15] to evaluate the tolerance of *S. cerevisiae* recombinant strains towards different inhibitors. Inhibitory growth conditions used in this study were 3.6 g/L acetic acid, 5 g/L acetic acid, 5 mM H_2_O_2_, 42 °C, 1.2 g/L 4-hydroxybenzaldehyde, 1.2 g/L phenol, 1.2 g/L vanillin, 1.5 g/L syringaldehyde, 58 g/L NaCl, 3 g/L propionic acid, and 0.22 g/L sorbic acid, respectively.

The content of intracellular ROS and activities of two antioxidant enzymes, namely, catalase (CAT) and superoxide dismutase (SOD), were estimated as described elsewhere [15].

Concentrations of IMP, adenosine 5′-triphosphate (ATP), adenosine 5′-diphosphate (ADP), and AMP were extracted as described previously [50]. Subsequently, the resultant extract was determined according to the previous study with minor modifications [51]. In brief, HPLC equipped with a Luna^®^ 5u C18 (2) 100 Å LC column (250 × 4.6 mm) was used and temperature was kept at 25 °C. Mobile phase A consisted of 0.1 M potassium phosphate buffer (pH 7.0), and mobile phase B consisted of pure acetonitrile. The pump was programmed to generate the following gradient: 0 min 100% A; 10 min 95% A and 5% B; 12 min 95% A and 5% B; 14 min 80% A and 20% B; 15.3 min 75% A and 25% B; and 16 min 100% A. The flow rate was adjusted to 0.6 mL/min. Peaks were identified and analyzed with a UV detector at λ_254_ nm.

To analyze intracellular amino acids content, yeast cell samples (50 mL) in the log phase were harvested by centrifuged at 3000*g* for 5 min at 4 °C. The cells were washed with distilled water twice and then re-suspended with 4 mL of 0.1 M hydrochloric acid. Cell extraction was prepared by vigorously vortex mixing with glass beads at 4 °C, and then centrifuged at 12,000*g* for 5 min at 4 °C. Supernatant was collected and supplemented with sulfosalicylic acid at a final concentration of 5% (w/v). The mixture was static at 4 °C for 1 h and then centrifuged at 15,000*g* for 30 min at 4 °C. Supernatant was adjusted to pH 1.7–2.2 with 1 M NaOH before detection. Intracellular amino acid concentration in the yeast cells was detected with High-Speed Aminoacid Analyzer (L-8900, Hitachi, Japan).

Yeast cells cultured in the fermentation medium with or without 5 g/L acetic acid addition were harvested at exponential phase for detection of intracellular zinc content. The methods of dissolving cells and detecting zinc content were adopted from our previous report [12].

### Statistical analysis

All experiments were performed in triplicates. The results of real-time quantitative PCR, enzyme activities and fermentation test were expressed as mean and standard deviation (SD). Student *t* test was used for statistical analyses with significant levels, *: *p *< 0.05, **: *p *< 0.01.

## Additional file

**Additional file 1: Table S1.** Yeast strains used in this study. **Table S2.** List of primers used for RT-qPCR analysis in this work. **Table S3.** List of primers used for plasmids and strains construction in this work. **Fig. S1.** Influence of acetic acid stress on transcription of *ADE17* in different *S. cerevisiae* BY4741 strains. **Fig. S2.** Comparison of stress tolerance of the engineered yeast strains with that of the control strain BHO under various stressful conditions. **Fig. S3.** Potential transcription factors regulating the *ADE* genes. **Fig. S4.** Growth curve of the recombinant strains under various conditions. **Fig. S5.** Detoxification of furfural and 5-HMF by the recombinant yeast strains. **Fig. S6.** Effect of *ADE* genes overexpression on intracellular energy level at stationary phase. **Fig. S7.** Effect of amino acids addition on yeast growth under acetic acid stress condition. **Fig. S8.** Comparison of acetic acid tolerance of the mutant strain ADE17_mZRE and the control strain. **Fig. S9.** Impact of the *ADE* genes overexpression on succinic acid production.

## Data Availability

The data sets analyzed during the current study are available from the corresponding author on reasonable request.

## Abbreviations

GABA: γ-aminobutyric acid
AXP: adenine-nucleotide pool
AMP: adenosine 5′-monophosphate
GMP: guanosine 5′-monophosphate
ADP: adenosine 5′-diphosphate
ATP: adenosine 5′-triphosphate
IMP: inosine monophosphate
ADE: ADEnine requiring
HMF: hydroxymethyl-2-furfural
ROS: reactive oxygen species
PCR: polymerase chain reaction
RT-qPCR: real-time quantitative PCR
DCW: dry cell weight
CAT: catalase
SOD: superoxide dismutase
GSH: glutathione
RNR: ribonucleotide reductase
ABC transporter: ATP-binding cassette transporter
FAP: furfural, acetic acid and phenol
RNA‑seq: RNA sequencing
OD: optical density
G6P: glucose-6-phosphate
3PG: 3-phosphoglycerate
Succ: succinate
AKG: a-ketoglutarate
Oac: oxaloacetate
AICAR: 1-(5′-phosphoribosyl)-5-amino-4-imidazolecarboxamide
SCAIR: 1-(5′-phosphoribosyl)-5-amino-4-(*N*-succinocarboxamide)-imidazole
CAIR: 1-(5-phospho-d-ribosyl)-5-amino-4-imidazolecarboxylate
TCA: tricarboxylic acid
PPRP: 5-phosphoribosyl diphosphate
SGD: Saccharomyces Genome Database
YEASTRACT: Yeast Search for Transcriptional Regulators And Consensus Tracking
MIPS: The Munich Information Center for Protein Sequences
KEGG: Kyoto Encyclopedia of Genes and Genomes
ZRE: zinc-responsive element
