# Supplementary material for: Enhanced acetic acid stress tolerance and ethanol production in Saccharomyces cerevisiae by modulating expression of the de novo purine biosynthesis genes
Source: Biotechnol Biofuels. 2019 May 10;12:116. doi: 10.1186/s13068-019-1456-1 (PMC6509782; doi:10.1186/s13068-019-1456-1)
Supplement: Supplementary file 1 — Additional file 1: Table S1. Yeast strains used in this study. Table S2. List of primers used for RT-qPCR analysis in this work. Table S3. List of primers used for plasmids and strains construction in this work. Fig. S1. Influence of acetic acid stress on transcription of ADE17 in different S. cerevisiae BY4741 strains. Fig. S2. Comparison of stress tolerance of the engineered yeast strains with that of the control strain BHO under various stressful conditions. Fig. S3. Potential transcription factors regulating the ADE genes. Fig. S4. Growth curve of the recombinant strains under various conditions. Fig. S5. Detoxification of furfural and 5-HMF by the recombinant yeast strains. Fig. S6. Effect of ADE genes overexpression on intracellular energy level at stationary phase. Fig. S7. Effect of amino acids addition on yeast growth under acetic acid stress condition. Fig. S8. Comparison of acetic acid tolerance of the mutant strain ADE17_mZRE and the control strain. Fig. S9. Impact of the ADE genes overexpression on succinic acid production. [file 13068_2019_1456_MOESM1_ESM.docx]

# Additional file 1

**Enhanced acetic acid stress tolerance and ethanol production in *Saccharomyces cerevisiae* by modulating expression of the ‘*de novo*’ purine biosynthesis genes**

Mingming Zhang^1^, Liang Xiong^3^, Yajie Tang^2,5^, Muhammad Aamer Mehmood^1,6^, Zongbao (Kent) Zhao^4^, Fengwu Bai^1^, Xinqing Zhao^1*^

*^1^State Key Laboratory of Microbial Metabolism & School of Life Sciences and Biotechnology, Shanghai Jiao Tong University, Shanghai 200240, China*

*^2^Key Laboratory of Fermentation Engineering (Ministry of Education), Hubei Provincial Cooperative Innovation Center of Industrial Fermentation, Hubei Key Laboratory of Industrial Microbiology, Hubei University of Technology, Wuhan 430068, China*

*^3^School of Life Science and Biotechnology, Dalian University of Technology, Dalian 116024, China*

*^4^Department of Biotechnology, Dalian Institute of Chemical Physics, Chinese Academy of Sciences, Dalian 116023, China*

*^5^State Key Laboratory of Microbial Technology, Shandong University, Qingdao 266237, China.*

*^6^Department of Bioinformatics & Biotechnology, Government College University Faisalabad, Faisalabad 38000, Pakistan*

**Corresponding author, email: xqzhao@sjtu.edu.cn. Tel: +021-3420-6673*

# Additional tables

Table S1 Yeast strains used in this study

| Strains | Properties | Source/reference |
| --- | --- | --- |
| S288C | *S. cerevisiae* laboratory strain, *MATα SUC2 gal2 mal2 mel flo1 flo8-1 hap1 ho bio1 bio6* | Lab preservation |
| BY4741 | *S. cerevisiae* laboratory strain, *MATα his3-∆1 leu2-∆0 met15-∆0 ura3-∆0* | Euroscarf |
| Ethanol Red | Industrial bioethanol strain, MAT a/α | Fermentis, a division of L. I., Lesaffre, Lille, France. |
| SPSC01 | Wild-type, diploid, flocculate strain | Lab preservation |
| BHO | BY4741 carrying the empty pHO plasmid | This study |
| BADE1 | BY4741 with *ADE1* overexpression | This study |
| BADE13 | BY4741 with *ADE13* overexpression | This study |
| BADE17 | BY4741 with *ADE17*overexpression | This study |
| BYCas9 | BY4741 with plasmid pRS414_Cas9 | This study |
| ADE17_mzre | BY4741 with mutation of the ZRE in the *ADE17* promoter region | This study |

Table S2 List of primers used for RT-qPCR analysis in this work

| Primer name | Sequence (5’-3’) | Purpose |
| --- | --- | --- |
| rtACT1-F | GCCGAAAGAATGCAAAAGGA | RT-qPCR |
| rtACT1-R | GGAAGGTAGTCAAAGAAGCCAAGA | RT-qPCR |
| rtADE17-F | ATGATTCGTGACGCTGGTTTT | RT-qPCR |
| rtADE17-R | TTTTCACTCTACCTCCCAACATTTC | RT-qPCR |
| rtADE13-F | CGTGTTGGCTCCTTTGTCTT | RT-qPCR |
| rtADE13-F | CGCATTGGGTTTCTCTTGTAA | RT-qPCR |
| rtADE1-R | TAAGGAGAAGGGCATCATCA | RT-qPCR |
| rtADE1-R | GAGGAGTCTGGCGTTAGCAC | RT-qPCR |
| rtGSH1-F | TCCCTTTGACGCTGACTGTC | RT-qPCR |
| rtGSH1-R | AAACAGAGACCTGGAAGCGG | RT-qPCR |
| rtSOD1-F | TGTCTCTGCTGGTCCTCACTTC | RT-qPCR |
| rtSOD1-R | CACACCATTTTCGTCCGTCTT | RT-qPCR |
| rtCTT1-F | CGTTGTTTGCCACGCTTGTA | RT-qPCR |
| rtCTT1-R | GGCACTTGCAATGGACCAAG | RT-qPCR |

Table S3 List of primers used for plasmids and strain construction in this work

| Primer name | Sequence (5’-3’) |
| --- | --- |
| ADE17F | ATCCCGGGATGGCCAATTACACAAAAACCGCAATCCT |
| ADE17R | CCTTAATTAACTAATGGTGG AACAAACGGA TTGGGT |
| ADE1F | ATCCCGGGATGTCAATTACGAAGACTGAACTGG |
| ADE1R | CCTTAATTAATTAGTGAGACCATTTAGACCCTGTC |
| ADE13F | ATCCCGGGATGCCTGACTATGACAATTACACTAC |
| ADE13R | CCTTAATTAACTAAACATTTAACTTGACTTGTTCATCG |
| Donor ADE17zre | GGCGGGCAGCATACGCAATTTGAATTTGCAGTTTTTGACTCTTTTTTCCTTAGCGGAACGAACAATATTAATCATGCCAACTAAGTCATTGCAGAAGTCAACTTATCATTTATAAAGAAGATTCTACC^CTACAATGTGTGTACCCTGATGTGCCCAGAAATGAATTGACTCCTCTCATAACTTGTTAATATTCGGATGTGCATATCACGTTCGGATCTATGTATATAAATGTACGCTCGTTTTAAAATTTAGTGTAGTACCTAAAATCTTTCTTACCAGCAACTAATAGCCCTTGAAGTAGTTTTGCTAGCTTGGACATCAAAGCACATATCACCATCAAATGCGG |
| Table S3 cont. |  |
| gRNA_ADE17-F | GATCGGGTACACACATTGTAGGGT |
| gRNA_ADE17-R | AAACACCCTACAATGTGTGTACCC |
| ADE17-C-F | GGCAGCATACGCAATTTGAATT |
| ADE17-C-R | ATTTGATGGTGATATGTGCTTTG |
| PKanR | AGCCGTTTCTGTAATGAAGGA |
|  |  |

# Figure legends

**Fig. S1** Influence of acetic acid stress on transcription of *ADE17* in different *S. cerevisiae* strains*.*

A, Growth curve of yeast strains BY4741, SPSC01 and Ethanol Red (ER) under acetic acid stress or control conditions. Red spots represent the sampling time for RT-qPCR analysis. B, Effect of acetic acid on relative transcription level of *ADE17* in different yeast strains.

**Fig. S2** Comparison of stress tolerance of the engineered yeast strains with that of the control strain BHO under various stressful conditions.

Strains BADE1, BADE13, BADE17 and BHO were cultured in YPD agar medium with 3.6 g/L acetic acid (pH 3.7), 3.6 g/L acetic acid (pH 4.5), 2 mM sorbic acid, 1 M NaCl and 40 mM propionic acid addition, respectively. YPD agar medium without addition of any stressors was used as the control condition.

**Fig. S3** The potential transcription factors regulating the *ADE* genes.

A, Schematic diagram of the potential transcription factor binding sites in the promoter regions of *ADE1*, *ADE13* and *ADE17* genes; B, Potential regulation matrix among transcription factors of *ADE1*, *ADE13* and *ADE17* based on the YEASTRACT database.

**Fig. S4** Growth curve of the recombinant strains under various conditions.

Yeast cells BADE1, BADE13, BAEE17 and the control strain BHO were cultivated in the fermentation medium with 5 g/L acetic acid addition (pH 4.5) (A), 3.6 g/L acetic acid addition (pH 3.7) (B), no external stress addition (C) and mixed inhibitors (pH 4.5) (D) , respectively. Results are the average of three runs ± SD.

**Fig. S5** Detoxification of furfural and 5-HMF by the recombinant yeast strains.

Ethanol fermentation was performed in the bioreactor with simulated hydrolysate medium and pH controlled at 4.5. The concentrations of furfural and 5-HMF were detected by HPLC. The compositions of inhibitors were described in the main text.

**Fig. S6** Effect of *ADE* genes overexpression on intracellular energy level at stationary phase.

Samples of the recombinant yeast strains and the control strain BHO were harvested at stationary phase for determination of energy charge (A); intracellular ATP concentration (B); total AXP content (C) and intracellular IMP concentration (D). CK represents control condition without stress, AC represents acetic acid stress condition. Yeast cells were harvested at the stationary phase. Each value represented the mean value of three independent replicates. **p* value<0.05 and ***p* value<0.01 in significance analysis using *t* test.

**Fig. S7** Effect of amino acid addition on yeast growth under acetic acid stress condition.

*S. cerevisiae* BY4741 was cultured in the YPD medium with amino acids addition in the presence of 5.0 (A, B) or 3.6 (C, D) g/L acetic acid. A and C: glutamate (0, 0.5 and 1.5 g/L); B and D: glutamine (0, 0.5 and 1.5 g/L). Results are the average of three runs ± SD.

**Fig. S8** Comparision of acetic acid tolerance of the mutant strain ADE17_mZRE and the control strain.

Acetic acid tolernace of the mutant strain ADE17_mZRE was compared with that of the parent strain *S. cerevisiae* BY4741 through spot assay with or without acetic acid (5 g/L, pH 3.5) addition.

**Fig. S9** Impact of the *ADE* genes overexpression on succinic acid production.

Ethanol fermentation was performed in the bioreactor with 5 g/L acetic acid (A) or mixed inhibitors (B) supplemented and pH was controlled at 4.5. Succinic acid in the fermentation broth was determined by HPLC. Results are the average of three independent experiments.

**
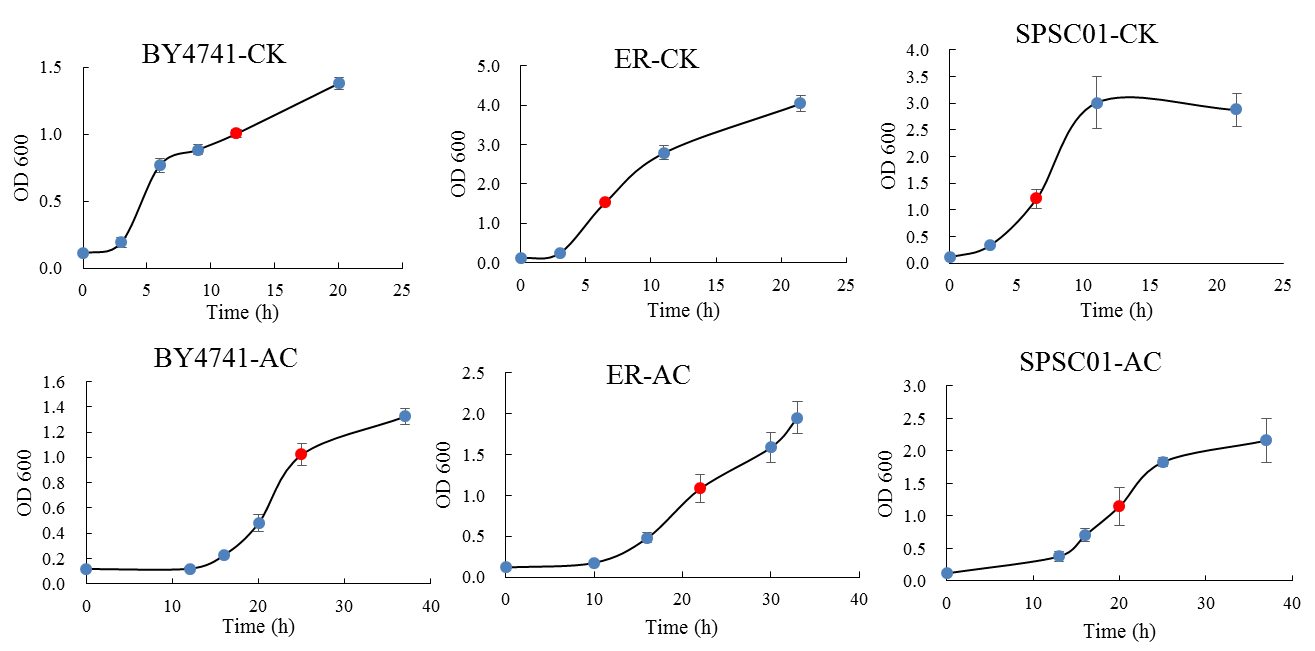
**

A

**Fig. S1**

**Fig. S2**

**A**

**B**

**Fig. S3**


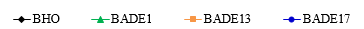


A B

3.6 g/L acetic acid

5 g/L acetic acid

Mixed inhibitors

Without inhibitors

C D

**Fig. S4**

**Fig. S5**

C

D

A

B

**Fig. S6**

A

B

**Fig. S7**

**Fig. S8**

**Fig. S9**
